# Wheat water productivity under saline irrigation in Northern China: a meta-analysis of effects and management practices

**DOI:** 10.3389/fpls.2026.1738026

**Published:** 2026-02-11

**Authors:** Xiaohe Jiang, Heli Cao, Ruopu Wang, Xinlong Li, Taisheng Du, Ling Tong, Jian Kang, Jia Gao, Risheng Ding

**Affiliations:** 1 State Key Laboratory of Efficient Utilization of Agricultural Water Resources, China Agricultural University, Beijing, China; 2 National Field Scientific Observation and Research Station on Efficient Water Use of Oasis Agriculture, China Agricultural University, Wuwei, Gansu, China; 3 Center for Agricultural Water Research in China, China Agricultural University, Beijing, China

**Keywords:** alternate saline and freshwater irrigation, saline irrigation, straw returning, water productivity, yield

## Abstract

**Introduction:**

Saline irrigation is an effective way to alleviate water scarcity in agriculture, but its productivity is constrained by salt stress. The effects of salinity on wheat yield and water productivity (WP), and how management practices can mitigate these effects, remain inadequately quantified.

**Methods:**

Through a meta-analysis of 2265 observations from field studies in Northern China, we quantified the responses of wheat yield, WP, and associated traits to saline irrigation and evaluated the efficacy of key management practices.

**Results:**

The wheat yield (-16.3%) and WP (-13.7%) were significantly reduced under saline irrigation. Salt stress primarily inhibited photosynthetic rate (Pn), which subsequently reduced leaf area index (LAI) and plant height (PH), ultimately restricting spike number (SN) and yield, while constraining WP. A salinity threshold of 5 g/L was identified, beyond which the declines in yield and WP became severe. Alternate saline and freshwater irrigation ameliorated stress, increasing PH (+12.1%), SN (+7.4%), yield (+5.9%), and WP (+13.1%). Similarly, straw returning increased SN (+11.3%), yield (+12.3%), and WP (+12.5%).

**Discussion:**

This study clarified the physiological cascade from salt stress to yield loss and confirm the critical role of the 5 g/L salinity threshold in sustaining wheat productivity. Alternate irrigation and straw returning mitigate salt stress through complementary pathways, enhancing plant growth and yield components. This meta-analysis provides evidence-based insights for optimizing saline irrigation management, supporting sustainable wheat production in water-scarce, salt-affected regions of northern China.

## 1 Introduction

Freshwater scarcity poses significant challenges to agricultural sustainability in Northern China. Saline irrigation has become a widespread practice to supplement limited freshwater resources (Wang, 2005; Sun et al., 2023). As the staple food ensuring regional food security (Chen and Zhang, 2023), wheat yields have decreased by over 25% in some areas under salt stress (Yuan et al., 2019). This poses a significant threat to self-sufficiency and national food security. Consequently, enhancing the yield and water productivity (WP) of wheat under saline water irrigation is crucial to safeguarding food production under the increasing water scarcity and soil salinisation.

Previous studies have conducted numerous experimental studies on the effects of salt stress on wheat, yet reported findings are often inconsistent. In terms of yield, Abbas et al. (2013) found that salt stress could reduce grain yield by 57.65%. However, Liu et al. (2016) found that the wheat yield did not significantly decline under brackish water irrigation. At the physiological level, Li et al. (2024) found that salt stress reduced photosynthetic rates (P_n_) and stomatal conductance (g_s_), while Pang et al. (2018) found that brackish water irrigation did not decrease P_n_ significantly. At the morphological level, Ma et al. (2010) found that salt stress led to a reduced plant height (PH), while the study of Shi et al. (2011) showed that it increased wheat PH instead. Under salt stress, both fresh weight and dry weight of wheat decrease with increasing salinity (Datta et al., 2009). Li et al. (2018) found that wheat yield decreased as the salinity of irrigation water increased. Due to differences in experimental designs and environmental conditions, the results of different studies vary, and most experimental studies focus on the quantification of results and lack analysis of influences. Therefore, it is essential to systematically quantify the responses of wheat yield, WP, and related traits to saline water irrigation, and clarify the underlying influences.

Meanwhile, a large number of studies on management practices capable of alleviating salt stress are also being continuously conducted. Alternative irrigation and straw returning were included in consideration, in which straw returning included straw mulching, chopped straw returning and straw-derived biochar incorporation. Zhai et al. (2019); Wang et al. (2023) revealed that alternating saline and freshwater irrigation could sustain wheat yield without significant mitigation under moderate to high salinity, while Kumar (2020) observed increased water productivity (WP) under saline conditions, though Cong et al. (2021) reported no significant WP improvement in salt−retentive soils. Zhang et al. (2019) found that straw returning could increase soil nutrients but decreased wheat yield; Chen et al. (2015) observed no significant yield impact under high salinity, and Li and Shi (2017) found no significant effect on WP under moderate salinity, whereas Lu et al. (2019) found that straw returning could improve WP and soil conditions, as well as counteract saline water yield loss. Overall, research on the underlying influences on which these treatments exert their effects remains limited, and consistent conclusions are lacking regarding their quantitative influence on wheat traits.

Meta-analysis is a quantitative analytical method used to integrate the results of multiple independent studies, addressing the issue of inconsistent conclusions from individual studies. This approach enhances the reliability and generalizability of results while identifying potential regulatory factors and their mechanisms (Hedges et al., 1999). At present, this method has been used in some studies for the analysis of crop P_n_ and yield responses under salt stress. For example, Cheng et al. (2021) utilized meta-analysis to quantify the responses of crop yield and WP under saline irrigation**;** however, there is no quantification of the improving effects of different management practices. Wang et al. (2025) conducted a meta-analysis on four crop species to investigate the responses of yield and WP to saline irrigation, along with the effects of different management practices. However, as their study did not focus specifically on wheat, the analysis remained limited in depth regarding this crop. Specifically, they examined only the general effects of saline irrigation on nitrogen use efficiency (NUE), water use efficiency (WUE), irrigation water use efficiency (IWUE), and yield in wheat, cotton, and maize. Cao et al. (2024) employed a meta-analysis to quantify the effects and interactions of drought and salt stresses on crop P_n_ and intrinsic water use efficiency (WUE_i_), but focused predominantly on the drought-salinity interaction mechanisms. Therefore, there remains a lack of in-depth research on the processes and trait interactions underlying the responses of wheat yield and water productivity (WP) to salt stress and its mitigation, particularly concerning yield components, leaf physiological traits, and growth traits.

In this study, we collected 2265 observations of wheat yield, WP and related traits under saline irrigation conditions in northern China, and conducted systematic quantification and analysis. The primary objectives of the study were: (1) to quantify the response of wheat yield, WP and related yield components, growth, and leaf physiological traits under saline irrigation; and (2) to analyze the different effects of management practices (salinity levels, alternate saline and freshwater irrigation and straw returning) on wheat yield and WP.

## 2 Materials and methods

### 2.1 Data assembly

To identify relevant studies regarding the responses of wheat under saline irrigation, we conducted an exhaustive search for studies published from 1997 to the present using the Web of Science (WOS) and China National Knowledge Infrastructure (CNKI). The search was performed using the search terms: (“saline irrigation” OR “brackish irrigation” OR “saline water” OR “brackish water” OR “saline water irrigation” OR “brackish water irrigation” OR “salt stress”) AND (“yield” OR “gain yield”) AND (“wheat” OR “Triticum aestivum”). The studies included in our meta-analysis were selected based on the following criteria: (1) the experimental was conducted on wheat; (2) the experiment was conducted in the field; (3) the experimental design included at least 2×2 full-factorial interactions, including treatment and control groups; (4) at least one trait related to crop yield, and WP was measured; (5) mean values (X), standard deviation (SD), and sample sizes (n) of traits in each treatment could be extracted from tables, figures and/or attachments. Following the above criteria, 34 published papers met our selection criteria (**Supplementary Figure S1**, **Supplementary Material S1**).

Data for the following wheat traits were extracted from each study: (1) yield, evapotranspiration (ET), WP, and irrigation water productivity (IWP); (2) yield component traits, including spike number (SN), number of grains per spike (SGN), and 1000-grain weight (GW); (3) growth traits, including leaf area index (LAI), PH, aboveground biomass (AGB) and harvest index (HI); (4) leaf physiological traits, including P_n_, transpiration rate (T_r_), and SPAD. Data from figures were extracted using the Web Plot Digitizer version 4.5 (https://apps.automeris.io/wpd4/). The collected data and other detailed information were listed in **Supplementary Table S1**, including 2265 observations for 18 wheat traits under saline irrigation. The salinity of soil in the harvest period of wheat and the amount of returned straw were also listed in **Supplementary Table S1**. Furthermore, the meteorological conditions of Hebei and Gansu, the main research sites in North China and Northwest China, are presented in **Supplementary Figure S2**.

We also extracted data on management practices (irrigation salinity level, irrigation method and straw returning) from 34 published papers. Irrigation salinity level was determined by the mineralization of irrigation water, which was classified as 1–3 g/L, 3–5 g/L, 5–7 g/L, and 7–9 g/L. Irrigation methods were classified as conventional saline irrigation, and alternate saline and freshwater irrigation. In addition, the data were classified as straw returning to the field and non-straw returning.

### 2.2 Data processing

After establishing a comprehensive database, if WP and IWP were missing, WP was calculated as follows (Equation 1):

(1) $$WP=\frac{Yield}{ET}$$

where WP is water productivity [kg/(ha·mm)], Yield is crop yield (kg/ha), and ET is wheat evapotranspiration (mm).

When ET were missing as well, it was calculated as follows (Equation 2):

(2) ET = P + I − ΔS

where ET is wheat evapotranspiration (mm); P is precipitation (mm); I is irrigation amount (mm); and ΔS is the change in soil water content in the 0–100 cm soil layer (mm).

IWP was calculated as follows (Equation 3):

(3) $$IWP=\frac{Yield}{I}$$

where IWP is irrigation water productivity [(kg/(ha·mm)], and I is irrigation amount (mm).

Some literature used electrical conductivity to measure the salinity of saline water. Thus, the mineralization degrees were calculated as follows (Equation 4) (Liu et al., 2013):

(4) M = 0.766×EC+109.89

where M is mineralization degree (g/L); and EC is electrical conductivity (dS/m).

While standard deviation data were not given in the literature, standard error (SE) was provided; SD (standard deviation) could be calculated by the formula (Equation 5):

(5) $$SD=SE\times \sqrt{n}$$

where n is the number of replications of the experiment; if neither SD nor SE was provided, the coefficient of variation (CV) was used to estimate the SD value. This was done by selecting data from the database where SD was not 0, and calculating the ratio of the standard deviation of the selected data points to their corresponding mean (m), i.e., calculating the CV, with the formula (Equation 6):

(6) CV=SD/m

The average coefficient of variation was obtained by averaging all the resulting CVs, and, for the data where SD was missing, the mean was ultimately used as an approximate substitute for the standard deviation by multiplying the mean by 1.25 times the average coefficient of variation. The formula was as follows (Equation 7):

(7) $$SD=1.25\bar{CV}\times m$$

To quantify the effects of salt stress on wheat traits, we used the natural logarithm of the response ratio Ln(RR) as a metric for effect size (Hedges et al., 1999), which was calculated as follows (Equation 8):

(8) $$Ln\left(RR\right)=ln\frac{x_{1}}{x_{2}}\;$$

where Ln(RR) denotes the logarithm of the response ratio, which represents effect size, $x_{1}$ denotes the mean value of the treatment group, and $x_{2}$ denotes the mean value of the control group.

The variance (v) of *Ln(RR)* was then calculated as follows (Equation 9):

(9) $$v=\frac{S_{1}^{2}}{n_{1}x_{1}^{2}}+\frac{S_{2}^{2}}{n_{2}x_{2}^{2}}\;$$

where n_2_ and n_1_ represent the number of replications for wheat traits under control and treatment, respectively, and s_2_ and s_1_ are the standard deviations of wheat traits under control and treatment.

### 2.3 Publication bias

The Rosenberg’s fail-safe-numbers (fsn) (Rosenberg, 2005), and funnel plots (Egger et al., 1997) were used to determine the reliability of the study. If the fsn exceeds 5k + 10 (where k represents the number of observations), it suggests high heterogeneity and no potential publication bias. If the P value of the Eggers’ test is > 0.05, the funnel plot is considered symmetric, i.e., there is no publication bias. Our results showed that for all traits, at least one of the two tests showed no publication bias (**Supplementary Figures S3-6**, **Supplementary Table S2**), that is, the study results included in the analysis are relatively comprehensive, no matter whether the results are positive or negative, there is a chance to be published and included in the analysis, so we did not consider publication bias to be an issue for the interpretation of the results (Doherty et al., 2021).

### 2.4 Statistical analysis

The “Metafor” package was used in the R software to calculate effect sizes. Positive effect sizes indicate positive effects on wheat traits, such as increased yield or P_n_, while negative values indicate negative effects. The effect was considered significant when the 95% confidence interval did not include 0. In addition, to clarify the regulatory influences on wheat yield and WP under saline irrigation, the regression analysis was performed using Origin 2025b software. P< 0.05 indicates a significant correlation between traits, and a larger R^2^ value indicates a stronger regulatory effect of the x-axis trait on the y-axis trait.

To reveal the significance of effects of different management practices on multiple wheat traits under saline irrigation, the analysis of variance (ANOVA) was used using the aov function in R software. Significant results are listed separately below the box plot, and P< 0.1 indicates that there is no significant effect of the management practice on the corresponding wheat traits. To clarify the significant differences among different subgroups of management practices, the multiple comparisons were conducted using the TukeyHSD function in R software, and the results of the multiple comparisons were represented by letters in the box plots.

## 3 Results

### 3.1 Overall responses of wheat traits to saline irrigation

**Figure 1:**
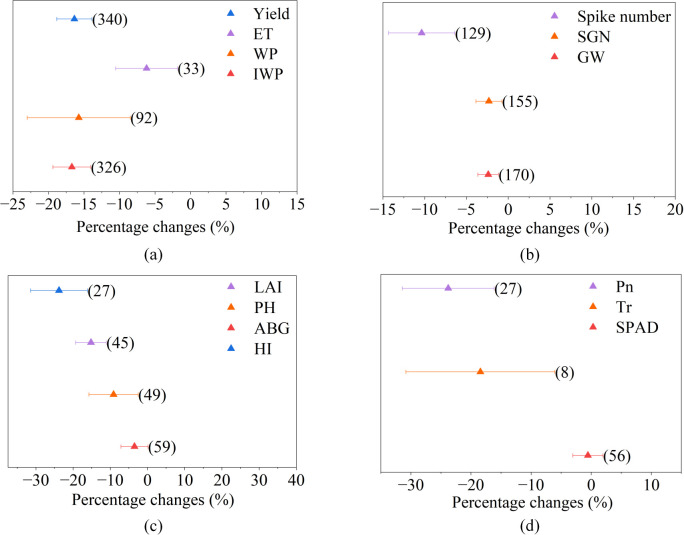
The percentage changes in **(a)** wheat yield, evapotranspiration (ET), water productivity (WP) and irrigation water productivity (IWP), **(b)** yield components, **(c)** growth, and **(d)** physiological traits under saline irrigation relative to freshwater irrigation. Points and bars represent the average response change and 95% confidence interval (CI). If the 95% CI does not overlap with zero, it is considered that saline irrigation has a significant impact on corresponding traits. Numbers in parentheses indicate the number of observations for each trait. SGN, number of grains per spike; GW, 1000-grain weight; LAI, leaf area index; ABG, aboveground biomass; HI, harvest index; P_n_, photosynthetic rate; T_r_, transpiration rate.

Meta-analysis showed that most traits decreased under saline irrigation (**Figure 1**). For the final output variables (**Figure 1A**), IWP showed the most substantial reduction (-16.7%), followed by yield (-16.3%), WP (-13.7%), and ET (-6.1%). For yield component traits (**Figure 1B**), the SN, GW, and SGN all experienced a significant decrease (-10.3%, -2.4% and -2.3%, respectively). For growth traits (**Figure 1C**), the LAI showed the largest decrease by -25.0%, followed by PH (-15.2%), AGB (-9.1%), and the HI (-3.5%), and the decrease in HI was not significant. For leaf physiological traits (**Figure 1D**), P_n_ decreased most substantially by -23.8%, followed by T_r_ and SPAD (-18.4% and -0.5%, respectively). The decrease in SPAD was not statistically significant.

### 3.2 Wheat yield and water productivity under saline irrigation

**Figure 2:**
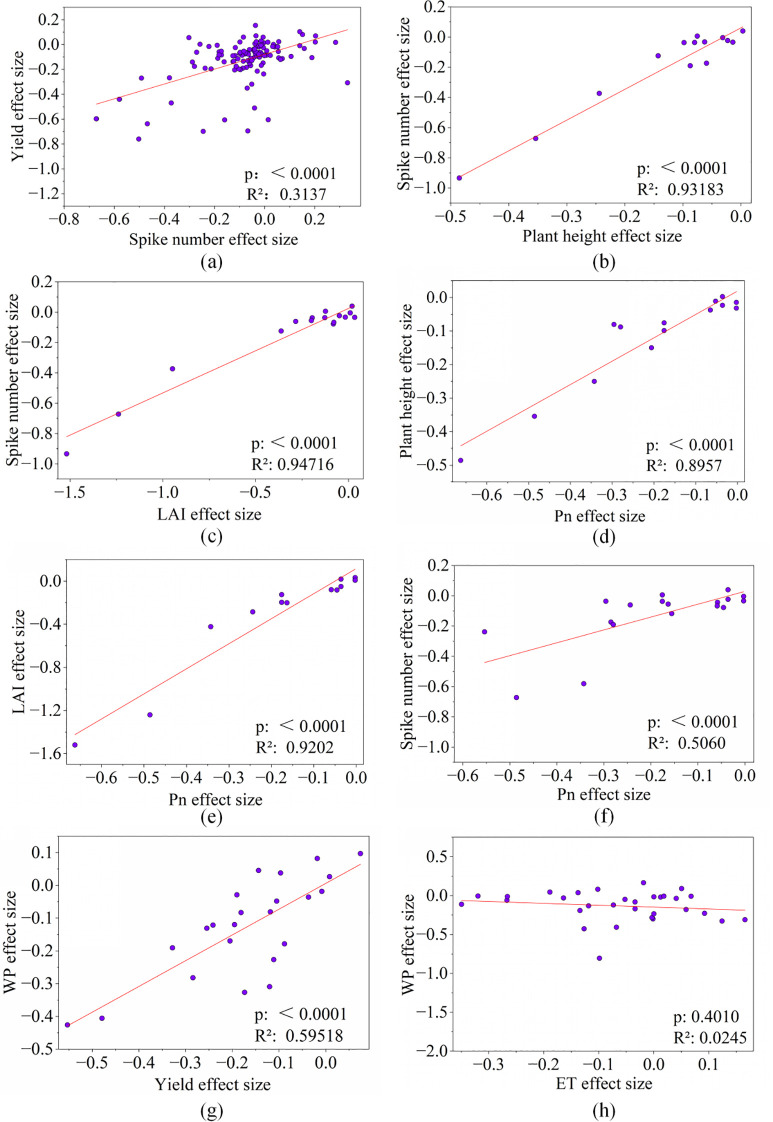
The correlation between the effect sizes of wheat yield, evapotranspiration (ET), water productivity (WP), yield components, growth and leaf physiological traits. **(a)** the correlation between the effect size of yield and spike number, **(b)** the correlation between the effect size of spike number and plant height, **(c)** the correlation between the effect size of spike number and plant height, **(d)** the correlation between the effect size of plant height and P_n_, **(e)** the correlation between the effect size of LAI and Pn, **(f)** the correlation between the effect size of spike number and P_n_, **(g)** the correlation between the effect size of WP and yield, **(h)** the correlation between the effect size of WP and ET. The correlations among the traits were considered significant at the significance level of p < 0.05, and R^2^ was the correlation coefficient. Abbreviations: LAI, leaf area index; P_n_, photosynthetic rate.

A significant positive correlation was observed between the effect size of wheat yield and the effect size of SN (R^2^ = 0.314, p< 0.0001) (**Figure 2A**). Subsequently, the effect size of SN was significantly correlated with the effect size of PH (R^2^ = 0.932, p< 0.0001) (**Figure 2B**) and LAI (R^2^ = 0.947, p< 0.0001) (**Figure 2C**).

Furthermore, the effect size of PH (R^2^ = 0.896, p< 0.0001) (**Figure 2D**) and LAI (R^2^ = 0.920, p< 0.0001) (**Figure 2E**) showed a significantly positively correlation with the effect size of P_n_; an additional significant positive correlation between the effect sizes of SN and P_n_ (R^2^ = 0.5060, p< 0.0001) (**Figure 2F**) was also showed in our results. Besides, we observed that the effect size of WP was significantly positively correlated with the effect size of yield (R^2^ = 0.595, p< 0.0001) (**Figure 2G**) rather than ET (**Figure 2H**) whose correlation was negative.

### 3.3 Effects of irrigation salinity levels on wheat traits

**Figure 3:**
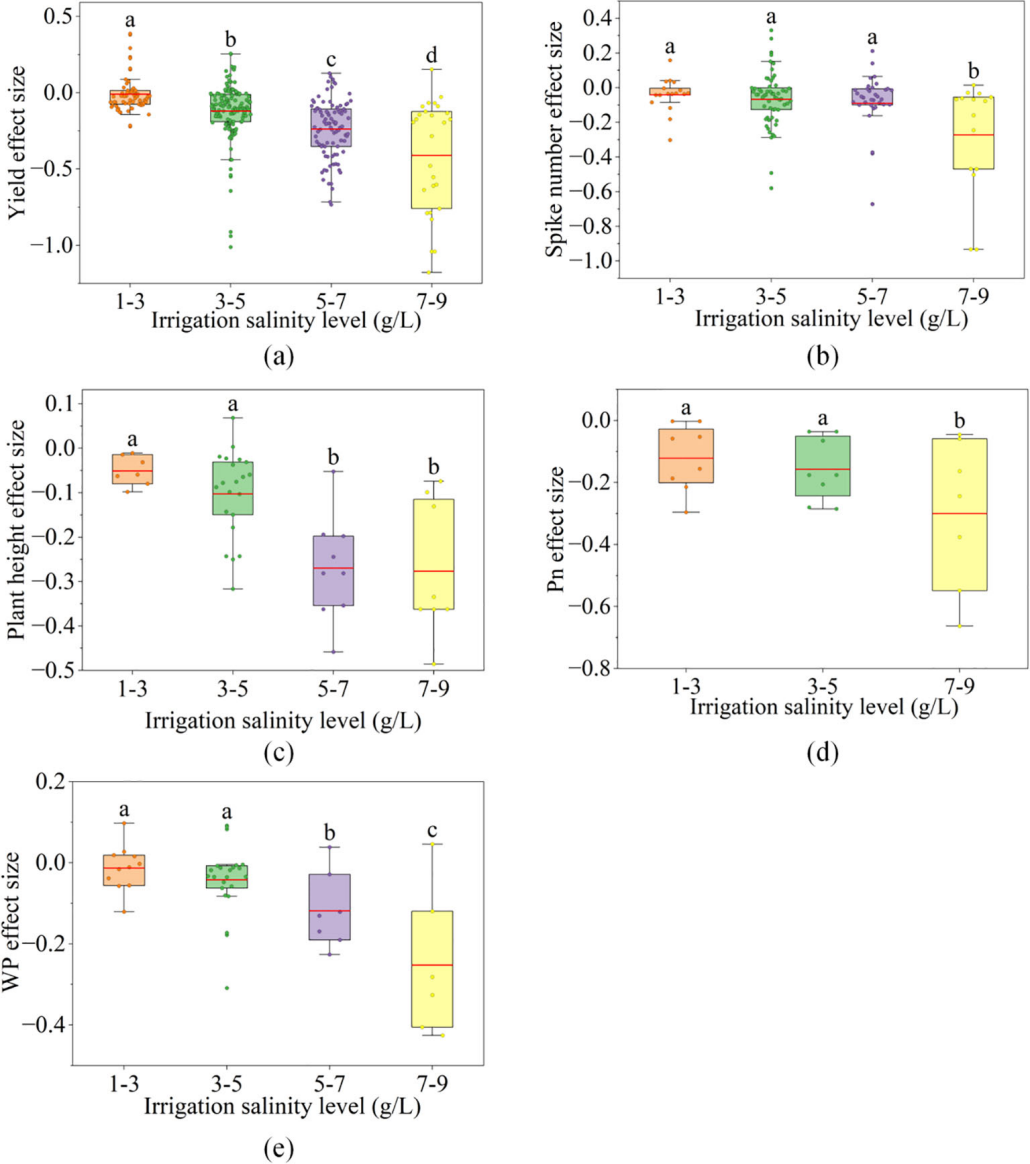
The box plots show the distribution of effect sizes of saline irrigation on wheat yield, spike number, plant height and water productivity (WP) at different salinity levels. **(a)** the distribution of yield effect sizes under saline irrigation, **(b)** the distribution of spike number effect sizes under saline irrigation, **(c)** the distribution of plant height effect sizes under saline irrigation, **(d)** the distribution of Pn effect sizes under saline irrigation, **(e)** the distribution of WP effect sizes under saline irrigation. The X-axes indicate irrigation salinity levels (g/L). Lowercase letters represent the results of multiple comparisons: there is no significant difference in effect sizes between groups with the same lowercase letter, while there is a significant difference in effect sizes between groups with different lowercase letters.

The effects of irrigation salinity levels on wheat traits were shown in **Supplementary Table S3**. The effects on wheat yield and WP were significant (**Supplementary Table S3**, p< 0.001), and the effect size of yield and WP decreased with increasing salinity level (**Figures 3A, E**). Compared with freshwater irrigation, when the salinity levels of saline irrigation were 1–3 and 3–5 g/L, the yield was decreased by 1.2% and 12.0% (**Figure 3A**), the WP was decreased by 1.3% and 4.2% (**Figure 3E**), both of which were relatively small (yield:<15%, WP:<5%). Correspondingly, for yield components, growth traits and physiological traits, the effects on wheat SN, PH and P_n_ were significant (**Supplementary Table S3**, p< 0.1). When the salinity levels were 1–3 g/L and 3–5 g/L, the SN was decreased by 4.2% and 6.7% (**Figure 3B**), the PH was decreased by 4.6% and 9.5% respectively (**Figure 3C**), and the P_n_ was decreased by 12.8% and 15.9% (**Figure 3D**); furthermore, there was no significant difference between the two salinity levels.

However, when the salinity levels of saline irrigation were 5–7 and 7–9 g/L, the extent of decline in yield and WP significantly increased, and the yield was decreased by 23.7% and 39.0% (**Figure 3A**), and the WP was decreased by 11.8% and 25.2% (**Figure 3E**). Correspondingly, for yield components traits, when the salinity level was 5–7 g/L, the SN was decreased by 9.3% (**Figure 3B**). When the salinity level was 7–9 g/L, the SN was decreased by 26.3% (**Figure 3B**), significantly greater than the degree of 5–7 g/L. For growth traits, when the salinity levels were 5–7 and 7–9 g/L, the PH was decreased by 27.3% and 27.9% (**Figure 3C**), significantly greater than the degrees of 1–3 and 3–5 g/L. For physiological traits, the P_n_ was decreased by 36.7% under the salinity level of 7–9 g/L (**Figure 3D**).

### 3.4 Effects of alternate saline and fresh irrigation on wheat traits

**Figure 4:**
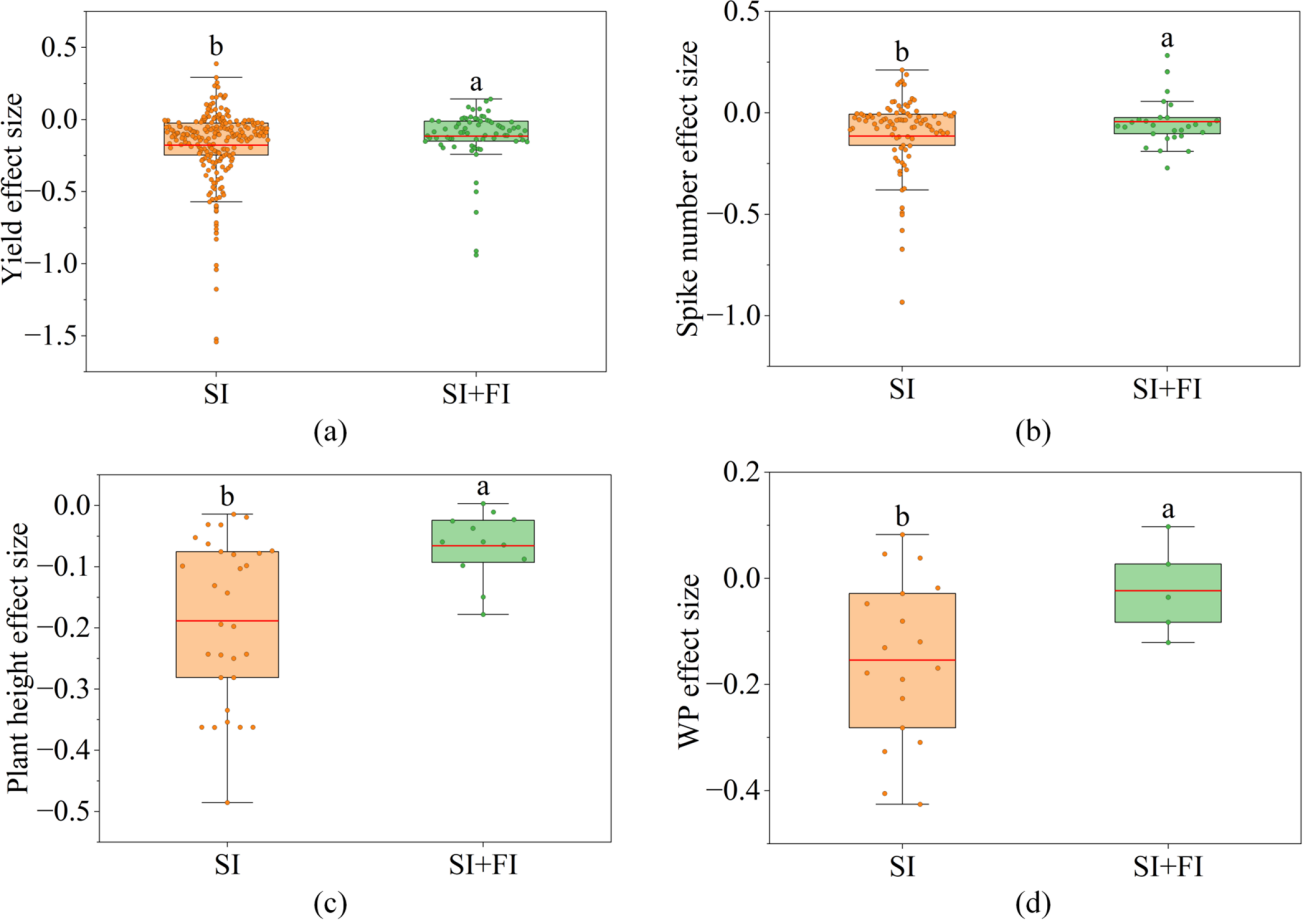
The box plots show the distribution of effect sizes of alternate saline and fresh irrigation on wheat yield, spike number, plant height and water productivity (WP) compared with conventional saline irrigation. **(a)** the distribution of yield effect sizes under alternate saline and fresh irrigation, **(b)** the distribution of spike number effect sizes under alternate saline and fresh irrigation, **(c)** the distribution of plant height effect sizes under alternate saline and fresh irrigation, **(d)** the distribution of WP effect sizes under alternate saline and fresh irrigation. The "SI" in the X-axes refer to conventional saline irrigation, while "SI + FI" refer to alternate saline and fresh irrigation. Lowercase letters represent the results of multiple comparisons: there is no significant difference in effect sizes between groups with the same lowercase letter, while there is a significant difference in effect sizes between groups with different lowercase letters.

The effects of alternate irrigation on wheat traits were shown in **Supplementary Table S4**. The effects on wheat yield and WP were significant (**Supplementary Table S4**, p< 0.1). Under conventional saline irrigation, the yield and WP of wheat were decreased by 17.4% and 15.4%, respectively (**Figures 4A, D**). However, under alternate saline and fresh irrigation, the yield and WP of wheat were decreased by 11.5% and 2.3%, respectively (**Figures 4A, D**).

Correspondingly, for yield components traits, the effect of alternate irrigation on wheat SN was significant (**Supplementary Table S4**, p< 0.1). Under conventional saline irrigation, the SN of wheat was decreased by 11.4% (**Figure 4B**). However, under alternate saline and fresh irrigation, the SN of wheat was decreased by 4.0% (**Figure 4B**). For growth traits, the effect of alternate irrigation on wheat PH was significant (**Supplementary Table S4**, p< 0.05). Under conventional saline irrigation, the PH of wheat was decreased by 18.1% (**Figure 4C**). However, under alternate saline and fresh irrigation, the PH of wheat was decreased by 6.0% (**Figure 4C**).

### 3.5 Effects of straw returning on wheat traits

**Figure 5:**
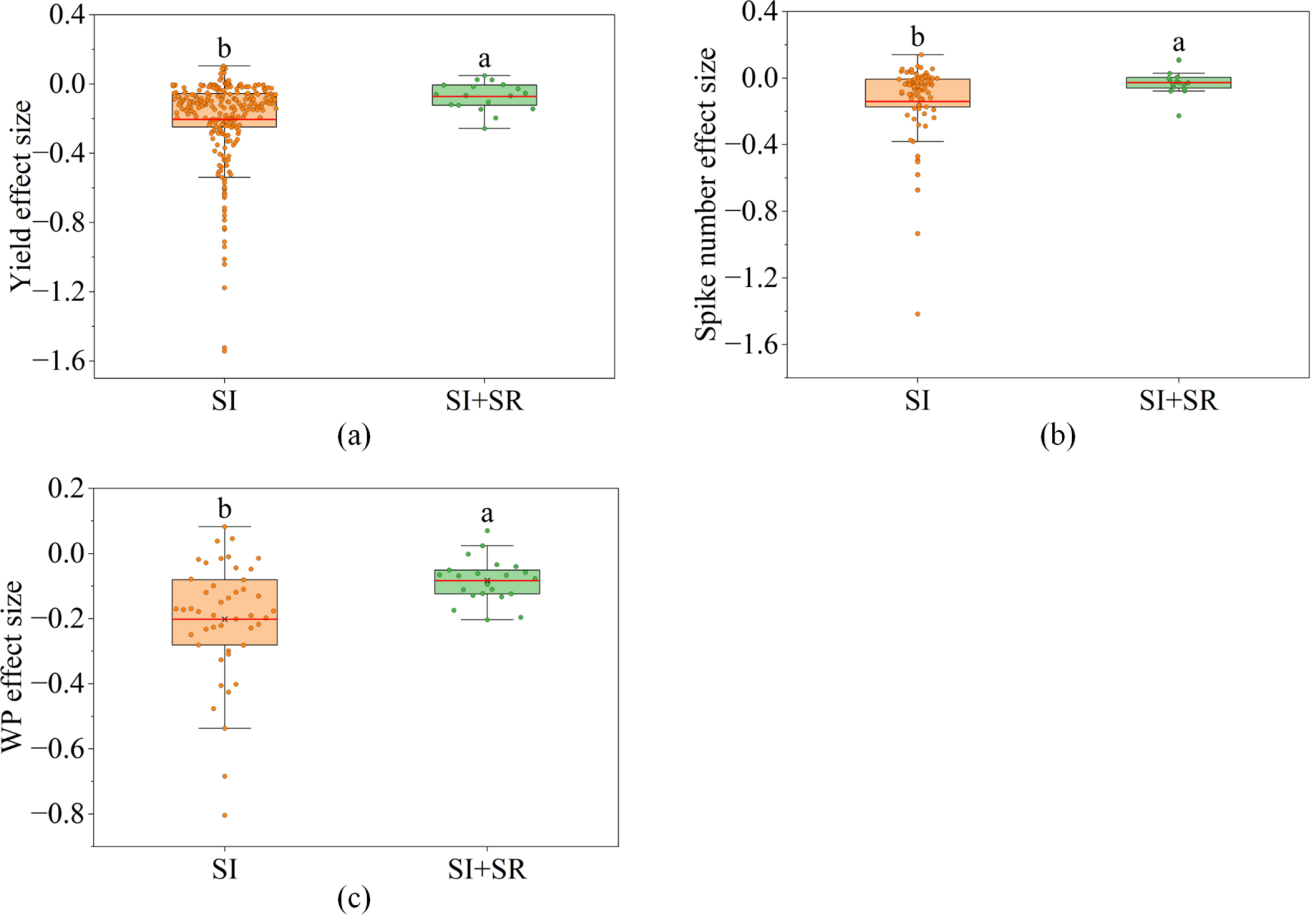
The box plots show the distribution of effect sizes of straw returning on wheat yield, spike number (SN) and water productivity (WP) under saline irrigation. **(a)** the distribution of yield effect sizes straw returning, **(b)** the distribution of spike number effect sizes straw returning, **(c)** the distribution of WP effect sizes straw returning. The "SI" in the X-axes refer to conventional saline irrigation, while "SI+SR" refer to saline irrigation combined with straw returning. Lowercase letters represent the results of multiple comparisons: there is no significant difference in effect sizes between groups with the same lowercase letter, while there is a significant difference in effect sizes between groups with different lowercase letters.

The effects of straw returning on wheat traits were shown in **Supplementary Table S5**, from which we could see that its effect on wheat yield, SN and WP were significant. For wheat yield and WP (**Supplementary Table S5**, p< 0.05), under saline irrigation, the yield and WP of wheat were decreased by 18.2% and 20.4%, respectively (**Figures 5A, C**). However, combined with straw returning, the yield and WP of wheat were decreased by 5.9% and 7.9%, respectively (**Figures 5A, C**).

Correspondingly, for yield components traits, the effect of straw returning on wheat SN was significant (**Supplementary Table S5**, p< 0.1). Under saline irrigation, the SN of wheat was decreased by 14.0% (**Figure 5B**). However, combined with straw returning, the SN of wheat was decreased by 2.7% (**Figure 5B**).

## 4 Discussion

**Figure 6:**
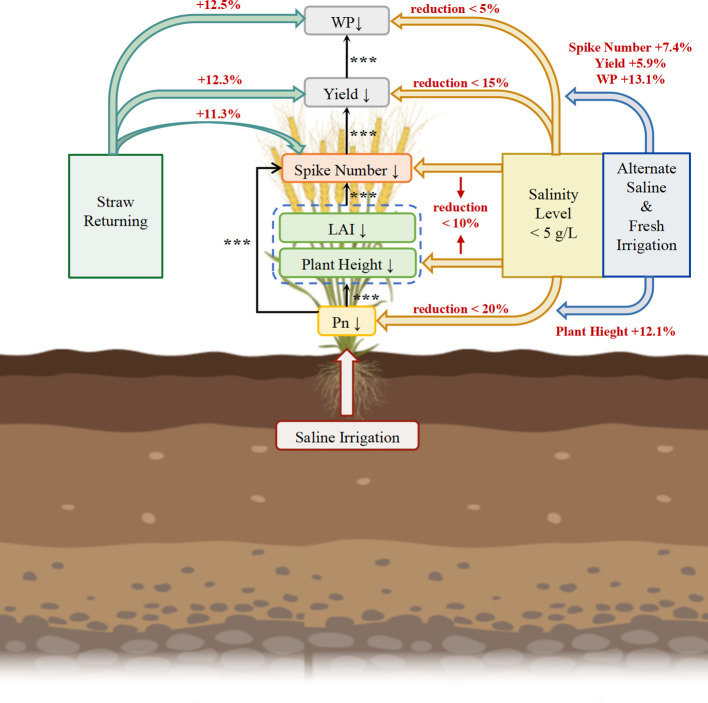
The schematic diagram of the responses of wheat yield and water productivity (WP) to saline water irrigation, as well as the improving effects of different management practices on wheat yield and WP. The black arrows point to the regulated traits at their ends. Significance levels (p< 0.001***) are shown. Management practices included irrigation water with irrigation salinity level, alternate saline and fresh irrigation, and straw returning. Yellow arrows point to wheat traits that maintained a high level at an irrigation salinity level of< 5 g/L. Blue arrows indicate traits boosted by alternate saline and fresh irrigation. Green arrows highlight traits improved through straw returning. The red text next to the arrows indicates the percentage increase in the corresponding traits achieved by the management practices. WP, water productivity; LAI, leaf area index; P_n_, photosynthetic rate.

This meta-analysis provided a systematic quantification of the influences on wheat yield and WP under saline irrigation, and evaluated the efficacy of improving management practices (**Figure 6**). Our findings delineated a clear physiological cascade from photosynthetic inhibition to yield loss, identified a critical salinity threshold of 5 g/L, and demonstrated that alternate irrigation and straw returning enhance productivity through distinct yet complementary effects.

### 4.1 Saline irrigation results in the physiological cascade from photosynthetic inhibition to yield loss

Saline irrigation triggers salt stress in plants, subsequently causing both osmotic stress and ion toxicity (Xu et al., 2011). Our results have shown that saline irrigation led to a reduction in wheat yield (-16.3%), WP (-13.7%) and IWP (-16.7%) (**Figures 1A**, **6**). Furthermore, the responses of yield-related components, growth traits, and leaf physiological traits in wheat to saline irrigation have been quantified (**Figures 1B-D**). Moreover, regression analysis has been employed to delineate the relationships between the effect sizes of yield and WP and other wheat traits, and further identify key regulatory pathways under salt stress (**Figures 2**, **6**). SN, SGN, and GW are the key yield component traits (Zhang et al., 2023b), among which SN is the most severely suppressed under saline irrigation (-10.3%) (**Figures 1B**, **6**). Additionally, yield loss is significantly correlated with SN (**Figures 2A**, **6**; **Supplementary Figure S7A, B**), underlying it is primarily driven by the suppression of SN, as salt stress primarily inhibits tiller initiation and survival and the subsequent development into productive ears (Wang et al., 2006; Liu et al., 2024), for the impaired assimilate translocation from vegetative organs to the spike (Wang et al., 1997).

In terms of growth traits, the significant decrease in both the LAI and PH (-25.0% and -15.2%, respectively) (**Figures 1C**, **6**) under saline irrigation indicates that salt stress severely inhibits cell elongation and leaf expansion, as well as overall photosynthetic production (Yang et al., 2011), which also leads to the decreased AGB in wheat (-9.1%) (**Figures 1C**, **6**) (Jaiswal et al., 2021). Furthermore, the results have shown significant correlations between LAI, PH and SN (**Figures 2B, C**, **6**), as reductions of PH and LAI diminish stem nutrient storage and plant competitiveness, which inhibit the energy and photosynthate availability for tillering, as well as the wheat tillering rate (Evers et al., 2006; Zhang et al., 2023b; Yin, 2024). For leaf physiological status, T_r_ and P_n_ significantly decreased by 18.4% and 23.8%, respectively (**Figures 1D**, **6**). Salt stress induces physiological drought in wheat, prompting the reduction of stomatal aperture (Wang et al., 2022), which decreases g_s_ (Soothar et al., 2021) and T_r_ (Zhao et al., 2020) and consequently diminishes P_n_ by limiting CO_2_ uptake (Pang et al., 2018). Furthermore, the decline in P_n_ is attributable to salt stress impairing photosystem II and key chloroplast photosynthetic enzymes activity (Vineeth et al., 2023). This reduction of P_n_ is significantly correlated to the decrease of PH and LAI (**Figures 2D, E**, **6**) by restricting photo assimilates, and subsequently reducing photosynthetic production (Hu et al., 2000; Zhou, 2021); meanwhile, it curtails the energy available for tiller development (Feng et al., 2023), which consists to the significant correlation between the effect sizes of wheat SN and P_n_ (**Figures 2F**, **6**). In addition, certain crops can sequester Na^+^ and Cl^-^ in vacuoles to maintain chloroplast ion homeostasis, avoiding ion interference with chlorophyll synthesis, and therefore SPAD has not shown a significant decrease (**Figures 1D**, **6**) (Robinson et al., 1983).

Thus, the yield loss under saline irrigation is driven by the suppression of P_n_ induced by salt stress, which limits SN directly as well as through inhibiting PH and LAI, and further leads to a significant yield reduction (**Figure 6**). Finally, for WP, its effect size under saline irrigation is significantly correlated with the effect size of yield, rather than with ET (**Figures 2G, H**, **6**), whose reduction is relatively small (6.1%) compared to the yield decline (16.3%, **Figure 1A**). The decoupling between the effect size of yield and ET (**Supplementary Figure S7l**) indicates that a certain level of water wheat maintains vital physiological processes under salt stress, but does not efficiently convert into yield (Feng et al., 2014). Under such resource-limited conditions, the ability to allocate more resources toward yield formation ultimately determines a higher WP in wheat (Zhang et al., 2023a).

### 4.2 A water salinity threshold is identified for wheat yield

The utilization of saline water irrigation can alleviate the scarcity of freshwater resources, with wheat being recognized as a moderately salt-tolerant crop (Guo et al., 2002; Wang et al., 2023). Our findings demonstrate that when the salinity of irrigation water ranges between 1–3 g/L, wheat yield and WP decrease by only 1.2% and 1.3%, respectively. At a higher salinity range of 3–5 g/L, the reductions in yield and WP are 12.0% and 4.2%, respectively (**Figures 3A, E**). These results indicate that moderate saline irrigation (< 5 g/L) does not lead to significant declines in wheat yield or WP (**Figure 6**).

P_n_, PH and SN were identified as key traits influencing wheat yield under saline irrigation (**Figures 2**, **6**). Our results further reveal that when irrigation water salinity is< 5 g/L, the reductions in both PH and SN are less than 10% (**Figures 3B, C**), while Pn is less than 20% (**Figure 3D**). Under low salinity level, the salt stress only induced stomatal closure, which can be partially compensated by the regulatory role of non-stomatal factors, thereby preventing a significant reduction in the P_n_ (Pang et al., 2018; Wang et al., 2022), and leading to maintaining PH and SN at a relatively high level. Furthermore, the study by Xu (2009) showed that under low salinity conditions, the increase in rhizomatous salinity allowed the crop to reduce the concentration of toxic ions in the cytoplasm by transporting excess Na^+^ and other toxic ions to the vesicles or tissues with low metabolic activity for storage. In addition, crops regulate osmotic pressure by isolating excess Na^+^ in vesicles through ionic compartments while accumulating proline, glycine betaine, etc (Polash et al., 2019). The regulation of osmotic pressure may affect the absorption of nutrients and water, which causes an effect on crop PH and SN, and ultimately reduces yield and WP (Yang et al., 2011). However, this regulation is not significant under salinity< 5 g/L (Wang et al., 2022). These findings provide strong support for the conclusion that the minimal decline in PH and SN under irrigation water salinity< 5 g/L is key to sustaining wheat yield and WP (**Figure 6**).

### 4.3 Alternate fresh and saline irrigation and straw returning can enhance wheat yield and water productivity

Compared to conventional saline irrigation, alternate fresh and saline irrigation can effectively leach soil salts and substantially alleviate the associated osmotic stress and ionic toxicity (Wang et al., 2023), thereby improving the root system’s capacity to absorb water and nutrients (Zhai et al., 2019). This enables the utilization of saline water resources while maintaining wheat yield and WP at relatively high levels (**Figure 6**), which consists with our results, that under conventional saline irrigation, wheat yield and WP decreased by 17.4% and 15.4%, respectively, whereas under alternate irrigation, the reductions decreased by 11.5% and 2.3% (**Figures 4A, D**). In terms of growth and yield component traits, PH and SN under alternate irrigation decreased by only 6.0% and 4.0%, respectively, representing improvements of 12.1% and 7.4% compared to conventional saline irrigation (**Figures 4B, C**). The study by Wu and Wang (2007); Bo et al. (2024) showed that alternate irrigation rapidly increases soil water potential, allowing stem cells to maintain adequate turgor pressure and enabling internodes to elongate following normal physiological rhythms, thereby reducing the decline in PH. Meanwhile, the reduction in soil salinity enhances nutrient availability for stem development (Pang et al., 2004; Zhang et al., 2017). Regarding SN, the alleviation of stress under alternate irrigation promotes tiller bud initiation and reduces tiller abortion, further contributing to improved yield and WP (Zhai et al., 2019; Wang et al., 2023). These findings support our conclusion that alternate fresh and saline irrigation enhances wheat yield and WP by mitigating the reductions in PH and SN (**Figure 6**).

Under saline irrigation, straw return creates a more favorable root zone environment by improving soil physical structure and regulating the water-salt balance (Lu et al., 2019). Our results have shown that compared to saline irrigation alone, straw returning increased wheat yield and WP by 12.3% and 12.5%, respectively (**Figures 5A, C**), demonstrating that it is an optimized management practice for enhancing wheat yield and WP under saline irrigation (**Figure 6**). SN is the key trait limiting wheat yield and wheat WP under saline irrigation (**Figures 2**, **6**). Further analysis revealed that compared to saline irrigation alone, straw return increased SN by 11.3% (**Figure 5B**). Zhang et al. (2022) reported that nutrients released from straw decomposition are more inclined to support tiller formation and spike development than seed filling of individual spikes. The finding supports our conclusion that under saline irrigation, straw return enhances wheat yield and WP primarily by improving SN (**Figure 6**).

This study provides new insights into the improvement of management practices, providing scientific support for improving yield and WP through management practices. However, the mechanistic analysis of saline irrigation impacts on wheat was limited by insufficient data and a lack of supporting physiological and biochemical traits (e.g., hydraulic properties, enzyme activities). Future work should include targeted field experiments to fill these critical data gaps.

## 5 Conclusion

The wheat yield and WP were decreased by 16.3% and 13.7% under saline irrigation, respectively. The primary underlying influence was identified as a physiological cascade: salt stress primarily inhibited P_n_, which subsequently reduced LAI and PH, ultimately restricted SN and yield while constraining WP. A critical salinity threshold of 5 g/L was established, beyond which the declines in yield and WP intensified markedly. When the irrigation salinity remained below this threshold, yield and WP reductions were contained within 15% and 5%, respectively. In terms of management strategies, alternate saline and freshwater irrigation increased the PH (+12.1%) and SN (+7.4%) of wheat, thereby further enhancing the yield (+5.9%) and WP (+13.1%). Similarly, straw returning increased the SN (+11.3%) of wheat, thereby further enhancing the yield (+12.3%) and WP (+12.5%). Based on these findings, it is recommended that in northern China, saline water irrigation should be limited to salinity levels not exceeding 5 g/L. Combining this with alternate fresh and saline irrigation and straw returning can further enhance yield and WP. These management measures help to achieve the goal of high water use efficiency in wheat production under the current situation of freshwater resource scarcity.

## Data Availability

The original contributions presented in the study are included in the article/**Supplementary Material**. Further inquiries can be directed to the corresponding author.
