# Supplementary material for: Wheat water productivity under saline irrigation in Northern China: a meta-analysis of effects and management practices
Source: Front Plant Sci. 2026 Feb 11;17:1738026. doi: 10.3389/fpls.2026.1738026 (PMC12933646; doi:10.3389/fpls.2026.1738026)
Supplement: Supplementary file 1 [file Supplementaryfile1.docx]

**Supplementary Information**

**Part 1 Supplementary figures:**


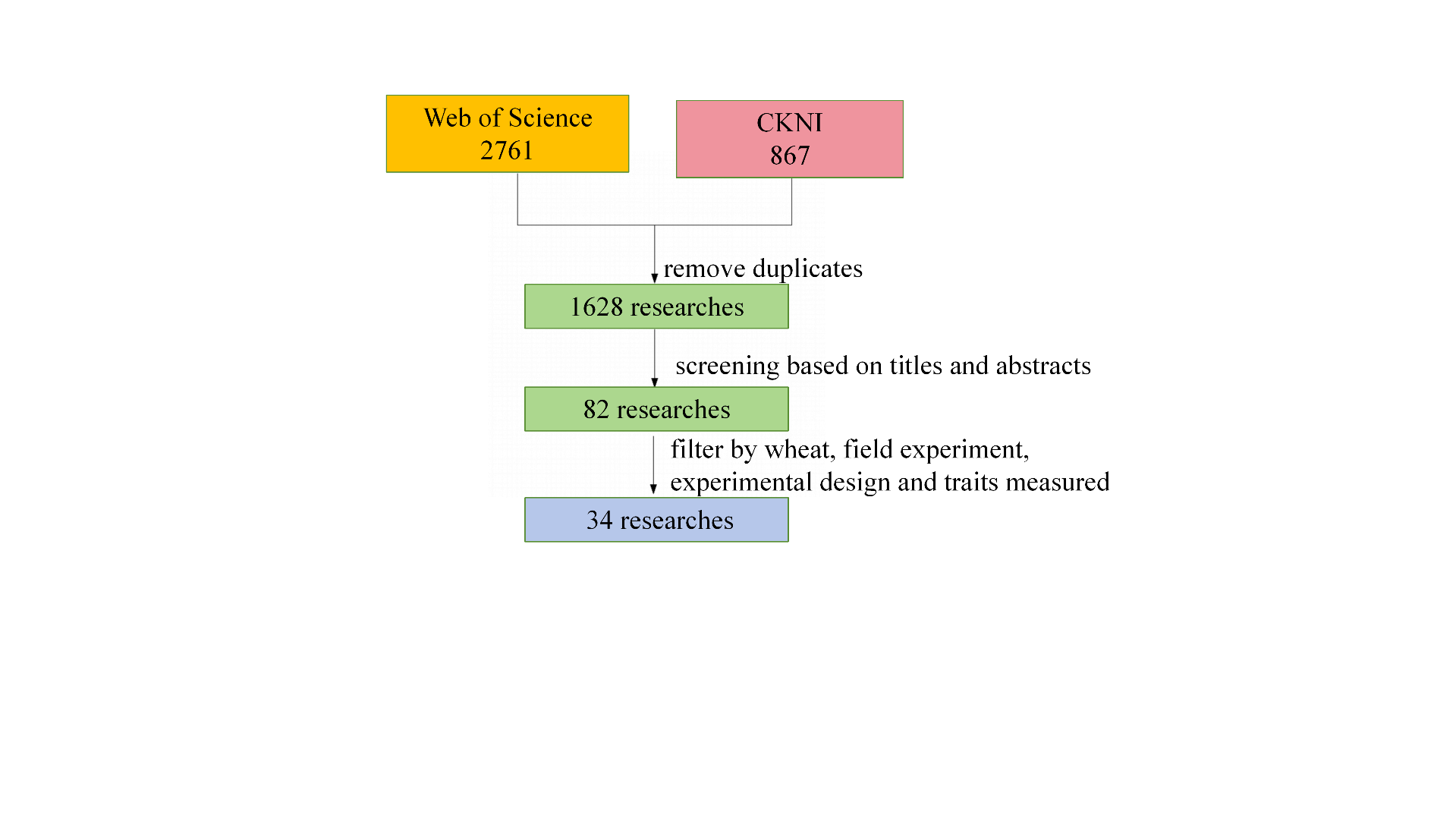


**Figure S1** The flowchart of research searching and screening.


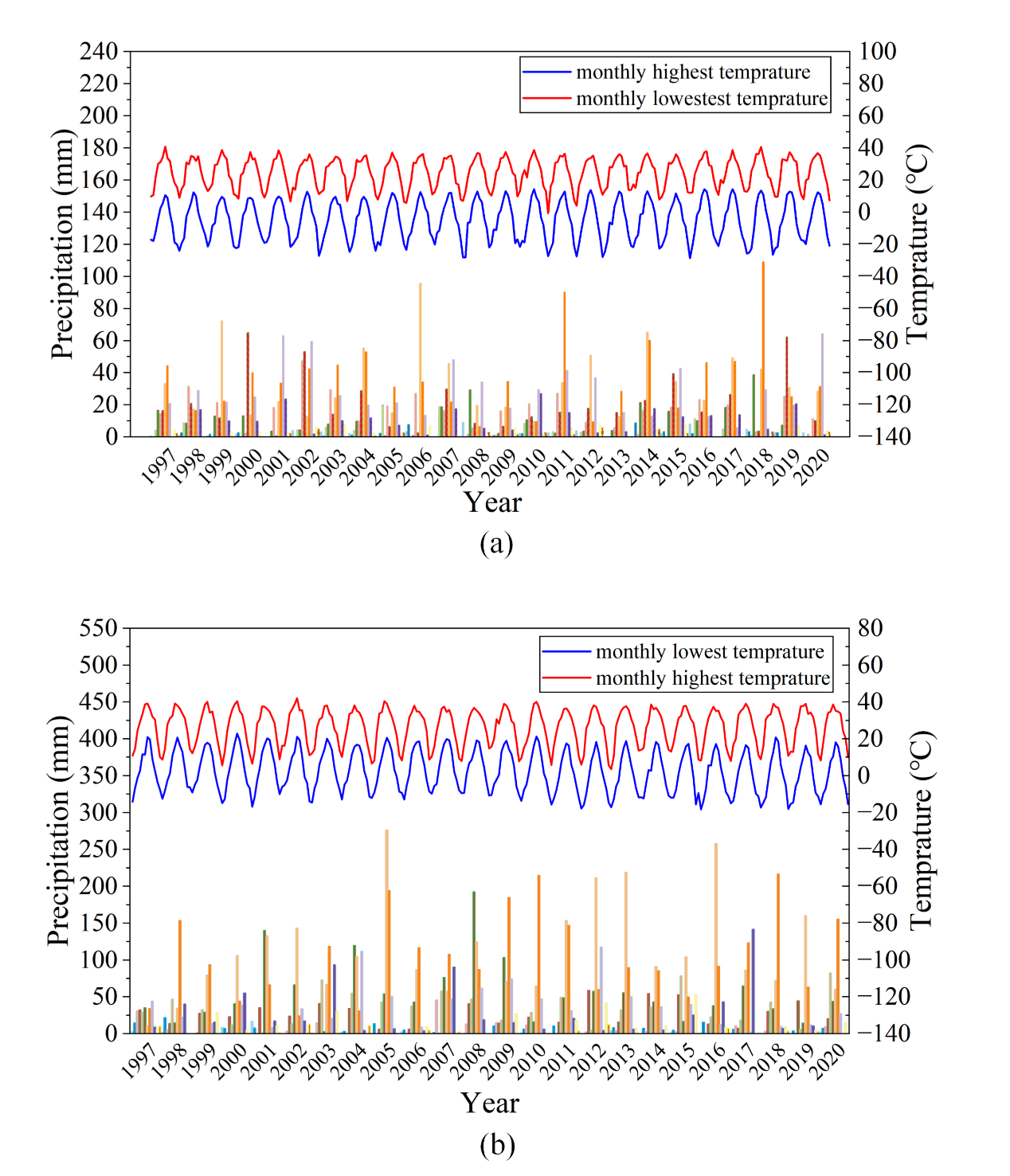


**Figure S2.** The meteorological conditions of Hebei and Gansu, the main research sites in North China and Northwest China, both including the monthly precipitation bar chart and the highest and lowest monthly temperature curve. (a) shows the meteorological conditions of Gansu; (b) shows the meteorological conditions of Hebei.


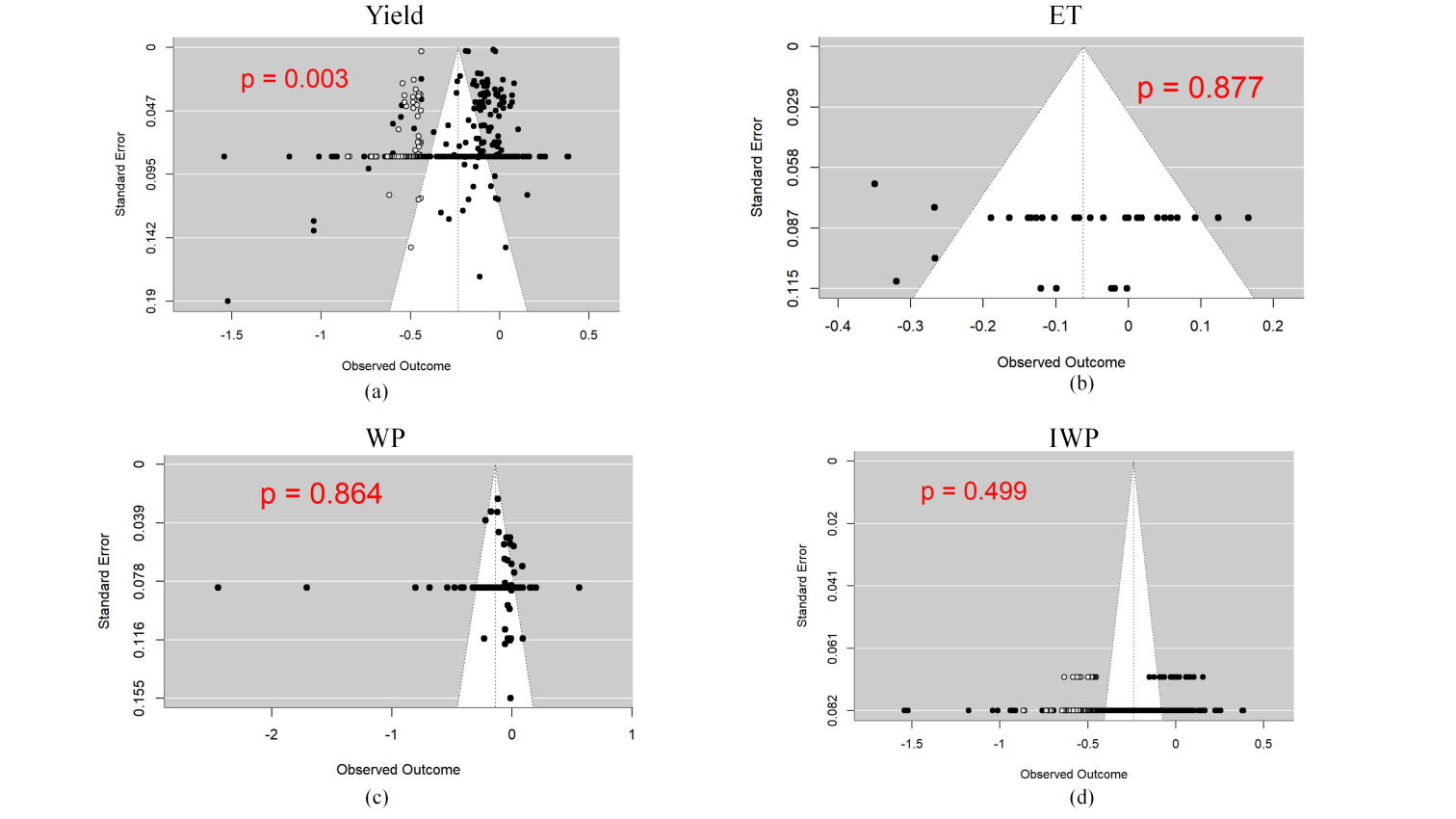


**Figure S3.** The funnel plots illustrating the possible effect of publication bias on wheat yield, ET, WP and IWP data under saline irrigation. (a) shows the funnel plots of wheat yield data; (b) shows the funnel plots of ET data; (c) shows the funnel plots of WP data; (d) shows the funnel plots of IWP data. The P-value in the figure represents the result of Egger’s regression test. Egger’s regression test was consistent with the data when the p-value was greater than 0.05. Abbreviations: ET, evapotranspiration; WP, water productivity; IWP, irrigation water productivity.


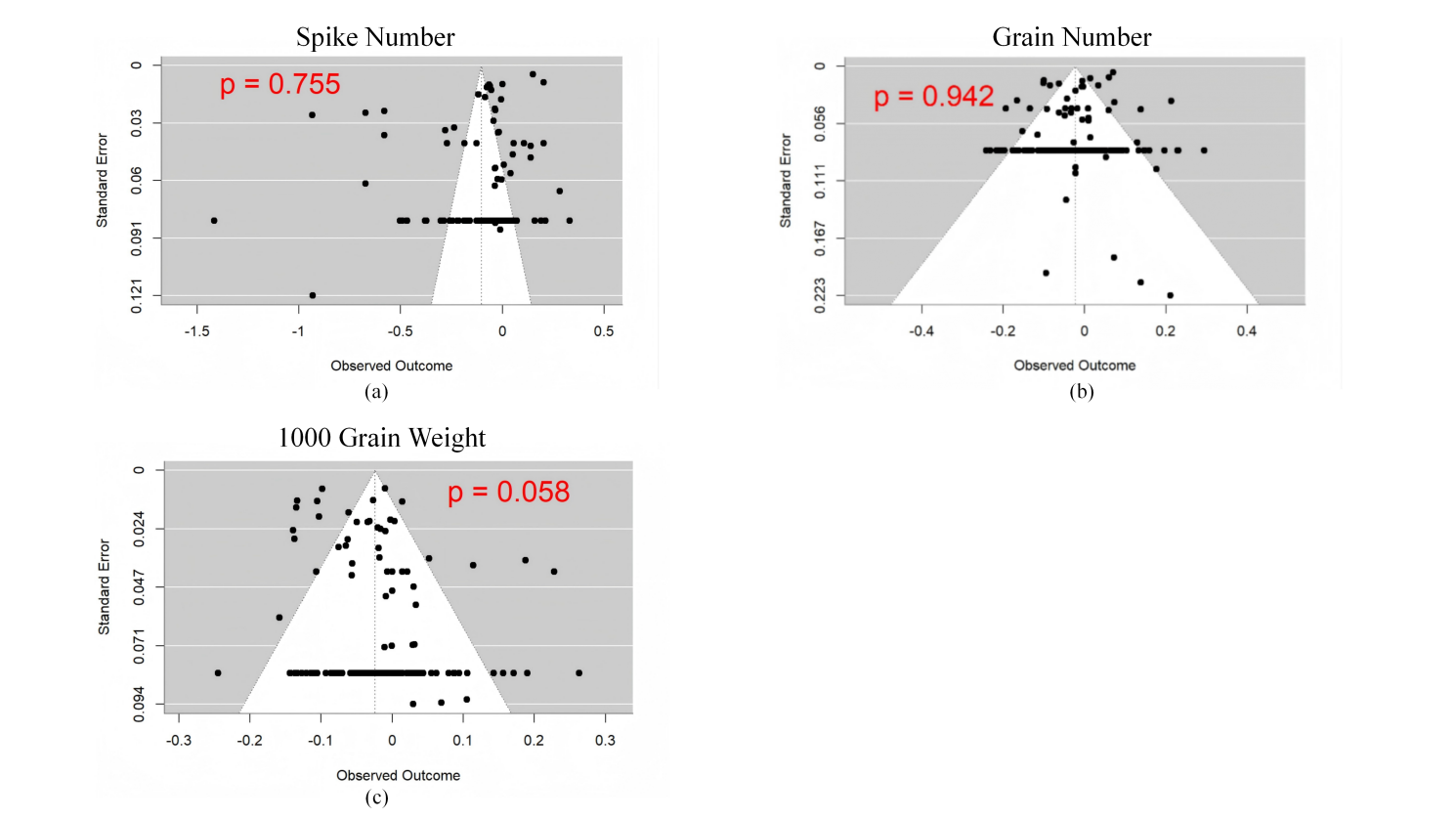


**Figure S4.** The funnel plots illustrating the possible effect of publication bias on wheat yield component data under saline irrigation. (a) shows the funnel plots of spike number data; (b) shows the funnel plots of the data of grain number per spike; (c) shows the funnel plots of 1000-grain weight data. The P-value in the figure represents the result of Egger’s regression test. Egger’s regression test was consistent with the data when the p-value was greater than 0.05.


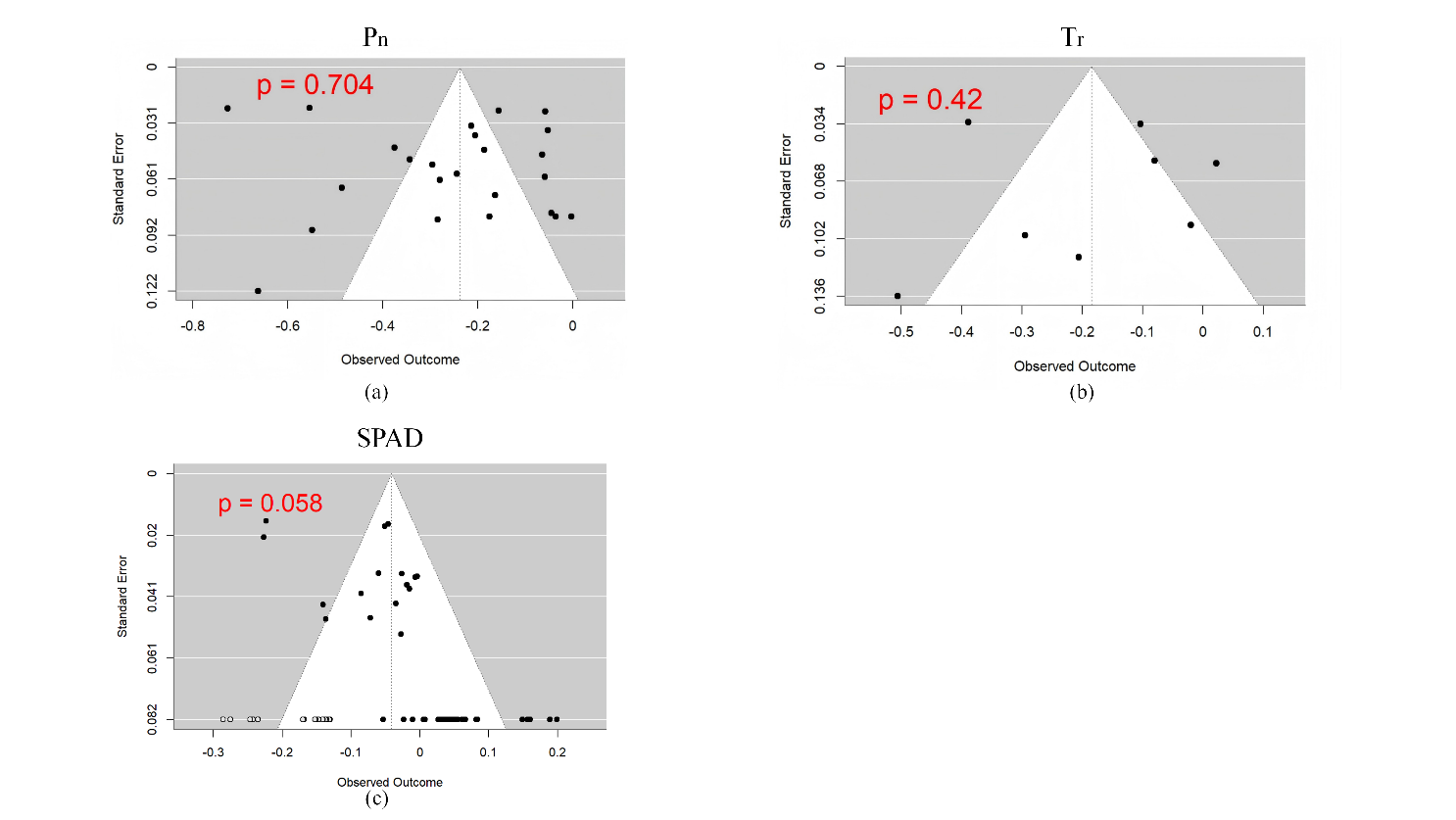


**Figure S5.** The funnel plots illustrating the possible effect of publication bias on physiological status data under saline irrigation. (a) shows the funnel plots of P_n_ data; (b) shows the funnel plots of T_r_ data; (c) shows the funnel plots of SPAD data. The P-value in the figure represents the result of Egger’s regression test. Egger’s regression test was consistent with the data when the p-value was greater than 0.05. Abbreviations: P_n_, photosynthetic rate; T_r_, transpiration rate; SPAD, chlorophyll component.


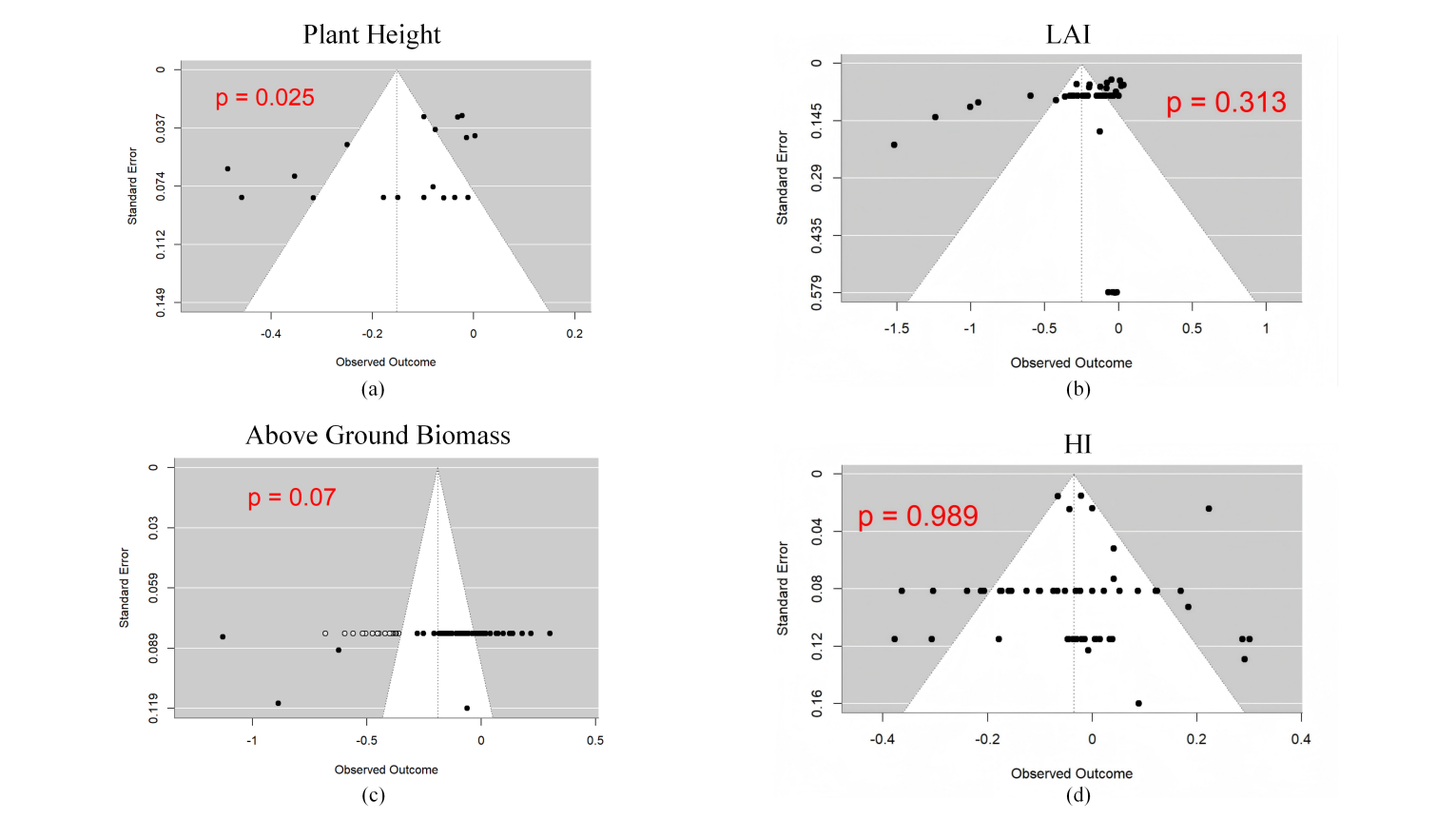


**Figure S6.** The funnel plots illustrating the possible effect of publication bias on growth morphology data under saline irrigation. (a) shows the funnel plots of plant height data; (b) shows the funnel plots of LAI data; (c) shows the funnel plots of above ground biomass data; (d) shows the funnel plots of HI data. The P-value in the figure represents the result of Egger’s regression test. Egger’s regression test was consistent with the data when the p-value was greater than 0.05. Abbreviations: LAI, leaf area index; HI, harvest index.


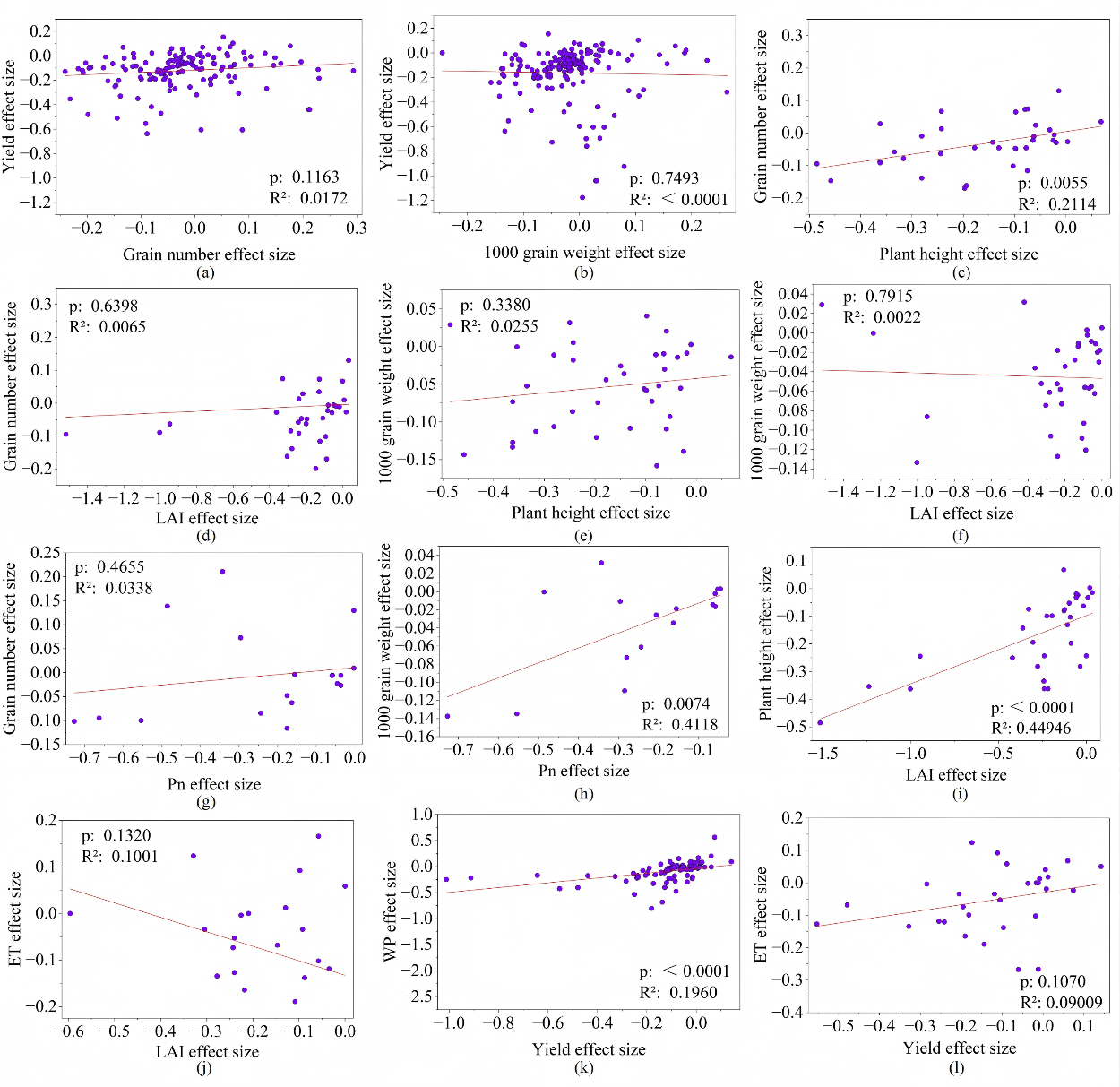


**Figure S7.** The correlation between the effect sizes of wheat yield, evapotranspiration (ET), water productivity (WP), yield components, growth and leaf physiological traits. The correlations among the traits were considered significant at the significance level of p < 0.05, and R^2^ was correlation coefficient. Abbreviations: LAI, leaf area index; P_n_, photosynthetic rate; ET, evapotranspiration; WP, water productivity.

**Part 2 Supplementary table:**

**Table S2.** The fail-safe N (FSN) value of wheat traits. If FSN is greater than 5k+10, it suggests that the meta-analysis result is unlikely to be substantially influenced by publication bias.

| Trait | yield | ET | WP | IWP | SN | SGN |
| --- | --- | --- | --- | --- | --- | --- |
| 5k+10 | 1560 | 175 | 470 | 1640 | 655 | 785 |
| FSN | 269156 | 181 | 10410 | 165262 | 18444 | 742 |
| Trait | GW | AGB | HI | LAI | PH | P_n_ |
| 5k+10 | 860 | 260 | 305 | 220 | 235 | 145 |
| FSN | 3472 | 939 | 140 | 4274 | 3309 | 7958 |
| Trait | T_r_ |  |  |  |  |  |
| 5k+10 | 50 |  |  |  |  |  |
| FSN | 216 |  |  |  |  |  |

Abbreviations: ET, evapotranspiration; WP, water productivity; IWP, irrigation water productivity; SN, spike number; SGN, number of grains per spike; GW, 1000-grain weight; AGB, aboveground biomass; HI, harvest index; LAI, leaf area index; PH, plant height; P_n_, photosynthetic rate; T_r_, transpiration rate.

**Table S3.** The significance of the effect of salinity levels on wheat traits under saline irrigation. When p is lower than 0.1, the impact of salinity levels on the corresponding trait is considered to be significant.

| Trait | Yield | SN | PH | Pn | WP | SGN |
| --- | --- | --- | --- | --- | --- | --- |
| p | <0.0001 | 0.0034 | <0.0001 | 0.0015 | <0.0001 | 0.1842 |
| Trait | GW | LAI | SPAD | HI | ET |  |
| p | 0.7537 | 0.5489 | 0.3088 | 0.9906 | 0.3868 |  |

Abbreviations: SN, spike number; PH, plant height; P_n_, photosynthetic rate; WP, water productivity; SGN, number of grains per spike; GW, 1000-grain weight; LAI, leaf area index; HI, harvest index; ET, evapotranspiration.

**Table S4.** The significance of the effect of alternate saline and fresh irrigation on wheat traits compared with conventional saline irrigation. When p is lower than 0.1, the impact of alternate saline and fresh irrigation on the corresponding trait is considered to be significant.

| Trait | Yield | SN | PH | WP | SGN | GW |
| --- | --- | --- | --- | --- | --- | --- |
| p | 0.0621 | 0.0782 | 0.0026 | 0.0703 | 0.1896 | 0.8825 |
| Trait | LAI | P_n_ | SPAD | HI | AGB | ET |
| p | 0.8918 | 0.4963 | 0.9978 | 0.8480 | 0.7034 | 0.1881 |

Abbreviations: SN, spike number; PH, plant height; WP, water productivity; SGN, number of grains per spike; GW, 1000-grain weight; LAI, leaf area index; P_n_, photosynthetic rate; HI, harvest index; AGB, aboveground biomass; ET, evapotranspiration.

**Table S5.** The significance of the effect of straw returning on wheat traits under saline irrigation. When p is lower than 0.1, the impact of alternate saline and fresh irrigation on the corresponding trait is considered to be significant.

| Trait | Yield | SN | WP | SGN | GW |
| --- | --- | --- | --- | --- | --- |
| p | 0.0226 | 0.0751 | 0.0026 | 0.4761 | 0.2432 |
| Trait | LAI | P_n_ | HI | AGB |  |
| p | 0.6141 | 0.1208 | 0.4000 | 0.7996 |  |

Abbreviations: SN, spike number; WP, water productivity; SGN, number of grains per spike; GW, 1000-grain weight; LAI, leaf area index; Pn, photosynthetic rate; HI, harvest index; AGB, aboveground biomass.

**Part 3 Supplementary text:**

**Text S1**

1. Cao, C., Zheng, C., Li, K., Dang, H., Li, W., and Ma, J. (2013). Impact of saline water irrigation with different salinities on yield and physiological indices of wheat. *Acta Automatica Sinica* 21(3). doi: 10.3724/sp.J.1011.2013.00347.
2. Chen, S., Shao, L., Sun, H., Zhang, X., and Li, Y. (2011). Effect of deficit irrigation with brackish water on growth and yield of winter wheat and summer maize. *Chinese Journal of Eco-Agriculture* 19(3)**,** 579-585. doi: 10.3724/sp.J.1011.2011.00579.
3. Chen, S., Shao, L., Sun, H., Zhang, X., and Li, Y. (2016). Effect of brackish water irrigation on soil salt balance and yield of both winter wheat and summer maize. *Chinese Journal of Eco-Agriculture* 24(08)**,** 1049-1058. doi: 10.13930/j.cnki.cjea.160075.
4. Cong, X., Pang, G.B., Xu, Z.H., and Wang, R.Z. (2021). Physiological Responses of Winter Wheat (Triticum Aestivum L.) to Alternate Irrigation with Fresh and Brackish Water. *Applied Ecology and Environmental Research* 19(4)**,** 3137-3152. doi: 10.15666/aeer/1904_31373152.
5. Dong, H., Li, M., Gu, Q., Han, W., Wu, D., Liu, Y., et al. (2021). Effects of Supplementary Irrigation with Brackish Water on Flag Leaf Senescence and Yield Components of Wheat in the Yellow River Delta. *Shandong Agricultural Sciences* 53(11)**,** 21-27. doi: 10.14083/j.issn.1001-4942.2021.11.004.
6. Gao, H., Fu, T., Tang, S., and Liu, J. (2023). Effects of saline water irrigation on winter wheat and its safe utilization under a subsurface drainage system in coastal saline-alkali land of Hebei Province, China. *Irrigation Science* 41(2)**,** 251-260. doi: 10.1007/s00271-023-00849-8.
7. Gong, Y., Sun, S., and Yan, H. (2017). Study on the Impact of Saline Water with Different Materialization Degree on Growth Characteristics and Yield of Winter Wheat. *Water Saving Irrigation* (09)**,** 33-37+42.
8. Gou, Q. (2021). *Effects of Irrigation Water with Different Salinity on Soil Physical and Chemical Properties and Growth of Winter Wheat.* Master Master's thesis, Shandong Agricultural University.
9. Guo, L., Zheng, C., Cao, C., Dang, H., Li, K., and Ma, J. (2017). Effect of Long-term Saline Water Irrigation on Photosynthetic Characteristics of Winter Wheat and Soil Salt Content. *Transactions of the Chinese Society for Agricultural Machinery* 48(01)**,** 183-190.
10. Huang, M., Zhang, Z., Zhai, Y., Lu, P., and Zhu, C. (2019). Effect of Straw Biochar on Soil Properties and Wheat Production under Saline Water Irrigation. *Agronomy* 9(8). doi: 10.3390/agronomy9080457.
11. Jiang, J., Huo, Z., Feng, S., and Zhang, C. (2012). Effect of irrigation amount and water salinity on water consumption and water productivity of spring wheat in Northwest China. *Field Crops Research* 137**,** 78-88. doi: 10.1016/j.fcr.2012.08.019.
12. Jiao, Y., Gao, W., Pan, Z., Li, K., and Shen, G. (2013). Effects of saline water irrigation on soil salt dynamics and yields of wheat and maize in low plain of Hebei province. *Agricultural Research in the Arid Areas* 31(02)**,** 134-140.
13. Jiao, Y., Wang, H., Zhang, S., Chen, W., and Zheng, C. (2021). Effects of sprinkling irrigation with brackish and fresh water mixing on yield of wheat and maize and movement of soil water and salt. *Agricultural Research in the Arid Areas* 39(06)**,** 87-94.
14. Li, G., Jiang, J., Ma, J., Zhang, J., and Guo, X. (2018). Effect of saline water irrigation on water - salt distribution and yield of wheat. *Journal of Drainage and Irrigation Machinery Engineering* 36(6)**,** 544-552. doi: 10.3969/j.issn.1674-8530.16.0296.
15. Liu, X., Feike, T., Chen, S., Shao, L., Sun, H., and Zhang, X. (2016). Effects of saline irrigation on soil salt accumulation and grain yield in the winter wheat-summer maize double cropping system in the low plain of North China. *Journal of Integrative Agriculture* 15(12)**,** 2886-2898. doi: 10.1016/s2095-3119(15)61328-4.
16. Ma, B., Wang, H., Liu, Y., Yang, T., and Gao, R. (2006). Influence of Brackish Water Irrigation on Growth and Yield of Winter Wheat in Saline Soil. *Water Saving Irrigation* (05)**,** 26-28.
17. Ma, J., Cao, C., Zheng, C., Li, K., and Zhang, C. (2010). Study on the Impact of Saline Water with Varied Mineral Content on Growth and Yield of Wheat. *Acta Agriculturae Boreali-Sinica* 25(S2)**,** 213-219.
18. Ma, Y., Dang, H., Li, K., Zheng, C., Cao, C., Zhang, J., et al. (2022). Effects of brackish water irrigation on grain quality characteristics and yield of winter wheat. *Chinese Journal of Applied Ecology* 33(04)**,** 1063-1068. doi: 10.13287/j.1001-9332.202204.003.
19. Mao, Z., Yu, Z., and Ma, Y. (2003). Influence of brackish water on the soil salt regime and yield of winter wheat and summer maize. *Journal of China Agricultural University* (S1)**,** 20-25.
20. Pang, H., Yang, J., and Yan, H. (2004). Effects of irrigation with saline water on soil salinity and crop yield. *Journal of Plant Nutrition and Fertilizers* (06)**,** 599-603.
21. Qiao, Y., Yu, Z., Zhang, Y., and Xin, J. (1999). Effects of Brackish Water Irrigation on Winter Wheat Growth and Soil Environmental Effects in Salinized Areas. *Soil and Fertilizer Sciences in China* (04)**,** 11-14.
22. Shao, Y., Li, Y., Sheng, F., Yan, Y., An, Y., and Zhang, Y. (2006). Safety of winter wheat and soil using brackish water irrigation. *Ecology and Environmental Sciences* (06)**,** 1241-1245. doi: 10.16258/j.cnki.1674-5906.2006.06.023.
23. Shi, L., Feng, Y., and Wang, H. (2024). Effects of Brackish Water Sprinkler Irrigation on the Photosynthetic Characteristics, Root Distribution and Yield of Winter Wheat. *Journal of Anhui Agricultural Sciences* 52(09)**,** 170-175+219.
24. Soothar, R.K., Zhang, W., Liu, B., Tankari, M., Wang, C., Li, L., et al. (2019). Sustaining Yield of Winter Wheat under Alternate Irrigation Using Saline Water at Different Growth Stages: A Case Study in the North China Plain. *Sustainability* 11(17). doi: 10.3390/su11174564.
25. Su, H., Sun, H., Dong, X., Chen, P., Zhang, X., Tian, L., et al. (2021). Did manure improve saline water irrigation threshold of winter wheat? A 3-year field investigation. *Agricultural Water Management* 258. doi: 10.1016/j.agwat.2021.107203.
26. Tian, M. (2023). Study on the Effects of Saline Water Irrigation on Soil Salt Distribution and Wheat Growth and Development. *Hebei Water Resources* (02)**,** 36-38.
27. Wang, T., Xu, Z., and Pang, G. (2019). Effects of Irrigating with Brackish Water on Soil Moisture, Soil Salinity, and the Agronomic Response of Winter Wheat in the Yellow River Delta. *Sustainability* 11(20). doi: 10.3390/su11205801.
28. Wang, Y., Pang, G., Fu, X., Yu, H., Zhang, L., Wang, X., et al. (2023). Effects of Alternate Irrigation with Brackish and Fresh Water on Physiological and Biochemical property and Yield of Winter Wheat. *Journal of University of Jinan(Science and Technology)* 37(02)**,** 163-171. doi: 10.13349/j.cnki.jdxbn.20230109.004.
29. Wei, B., Zhao, S., Niu, H., Xing, L., and Zhang, X. (1997). Experimental Study on Soil Salt Dynamics under Saline Water Irrigation. *Shanxi Hydrotechnics* (02)**,** 45-50.
30. Wu, Z. (2008). *Study on the Effect of Soil Water and Salt Distribution Characteristics and Winter Wheat Yield by Saline Water Border Irrigation.* doctoral thesis, Xi'an University of Technology.
31. Xu, J., Sun, W., Li, Y., Duan, M., Li, Z., and Yin, H. (2012). Effects of Straw Returning on Soil Salt Inhibition and Crop Yield under Brackish Water Supplementary Irrigation. *Soil and Fertilizer Sciences in China* (06)**,** 29-33.
32. Zhang, J., Jiang, J., Ma, J., Feng, S., and Li, G. (2016). An Experimental Study on the Effect of Different Irrigation Water and Salinity on Wheat Growth. *Water Saving Irrigation* (05)**,** 1-5+11.
33. Zhang, Y., and Lu, W. (2007). Affects of Brackish Water Irrigation on Output and Physiological Characters of Wheat. *Journal of Henan Agricultural Sciences* (08)**,** 31-34.
34. Zhang, Z., Zhang, Z., Feng, G., Lu, P., Huang, M., and Zhao, X. (2022). Biochar Amendment Combined with Straw Mulching Increases Winter Wheat Yield by Optimizing Soil Water-Salt Condition under Saline Irrigation. *Agriculture* 12(10). doi: 10.3390/agriculture12101681.
